# Dyslexia and configural perception of character sequences

**DOI:** 10.3389/fpsyg.2015.00482

**Published:** 2015-04-22

**Authors:** Joseph W. Houpt, Bethany L. Sussman, James T. Townsend, Sharlene D. Newman

**Affiliations:** ^1^Department of Psychology, Wright State UniversityDayton, OH, USA; ^2^Department of Psychological and Brain Sciences, Indiana UniversityBloomington, IN, USA

**Keywords:** capacity, dyslexia, configural processing, word superiority effect, individual differences

## Abstract

Developmental dyslexia is a complex and heterogeneous disorder characterized by unexpected difficulty in learning to read. Although it is considered to be biologically based, the degree of variation has made the nature and locus of dyslexia difficult to ascertain. Hypotheses regarding the cause have ranged from low-level perceptual deficits to higher order cognitive deficits, such as phonological processing and visual-spatial attention. We applied the capacity coefficient, a measure obtained from a mathematical cognitive model of response times to measure how efficiently participants processed different classes of stimuli. The capacity coefficient was used to test the extent to which individuals with dyslexia can be distinguished from normal reading individuals based on their ability to take advantage of word, pronounceable non-word, consonant sequence or unfamiliar context when categorizing character strings. Within subject variability of the capacity coefficient across character string types was fairly regular across normal reading adults and consistent with a previous study of word perception with the capacity coefficient—words and pseudowords were processed at super-capacity and unfamiliar characters strings at limited-capacity. Two distinct patterns were observed in individuals with dyslexia. One group had a profile similar to the normal reading adults while the other group showed very little variation in capacity across string-type. It is possible that these individuals used a similar strategy for all four string-types and were able to generalize this strategy when processing unfamiliar characters. This difference across dyslexia groups may be used to identify sub-types of the disorder and suggest significant differences in word level processing among these subtypes. Therefore, this approach may be useful in further delineating among types of dyslexia, which in turn may lead to better understanding of the etiologies of dyslexia.

## 1. Introduction

Developmental dyslexia is a neurobiologically based, lifelong learning disability that specifically affects the ability to read skillfully and is estimated to be present in 5–17.5% of children (Shaywitz, 1998). Reading deficits in dyslexia are considered unexpected and independent of factors such as intelligence and opportunity (see however Stanovich, 1996). There is no consensus on the etiology or core deficit in dyslexia and several theories have been proposed. It is generally associated with deficits in spelling, phonological/orthographical processing, rapid auditory processing, and short-term verbal memory (Ramus, 2003; Shaywitz and Shaywitz, 2005). Dyslexia has also been linked to other more domain general impairments such as automaticity (Nicolson and Fawcett, 2011), magnocellular functioning (Stein, 2001), and temporal auditory processing (Tallal, 1980). While phonological awareness has remained the most consistent explanatory marker (Ramus, 2003) of dyslexia, the cause of phonological impairment remains controversial. Dyslexia is often diagnosed in childhood and many dyslexic readers may build reading proficiency in adolescence and adulthood, however, reading often remains slow and effortful and there remains a phonological processing deficit (Wilson and Lesaux, 2001; Shaywitz and Shaywitz, 2005).

### 1.1. The word superiority effect and dyslexia

From the early days of experimental psychology, researchers have noted that normal reading adults are better at perceiving letters in the context of a word than alone or in random sequences (e.g., Cattell, 1886). Even when the informativeness of a word context is eliminated through careful experimental control (Reicher, 1969; Wheeler, 1970) normal reading adults perform better with a word context. The pervasive advantage is frequently referred to as the word superiority effect. The word superiority effect is a classical example of a configural superiority effect (cf. Pomerantz et al., 1977), but there is still some uncertainty as to the nature of the context advantage. Possible explanations have ranged from holistic processing of the word form (e.g., Healy, 1994) to independent processing of letters with some correction of letter level errors based on word level properties (e.g., Massaro, 1973; Pelli et al., 2003). Given that there is argument about the presence of a superiority effect, we focus on the degree of superiority rather than the locus of the superiority effect in this paper.

Given the robustness of the word superiority effect, one might inquire as to whether the effect is intact among individuals with developmental dyslexia. With dyslexia, reading is a generally slower and more effortful process. Potential loci of the reading deficit range from sub-word level, such as letter-phoneme correspondence (e.g., Blau et al., 2009; Blomert, 2011), to sentence level syntactic deficits. Tests of word superiority isolate one attribute of reading performance, and the extent to which individuals with dyslexia have a reduced or absent word superiority effect may be informative as to the nature of their deficits. Likewise, variation in the word superiority effect when comparing those with dyslexia and controls may also inform our understanding of the nature of the word superiority effect in the normal reading population.

Although research on dyslexia and the word superiority effect is limited, Grainger et al. (2003) have compared children with dyslexia and reading-age matched controls on the Reicher-Wheeler task (the standard paradigm for measuring the word superiority effect). Despite clear differences between the groups in ability to pronounce pseudowords, both groups were significantly better at identifying letters in the context of a word than in a non-word. The magnitude of the difference between words and non-words was nearly the same in both groups, and, if anything, slightly larger in the dyslexia group. This same basic effect was replicated by Ziegler et al. (2008), although they found statistically significant superiority effects in only response times, not accuracy.

Since the original demonstrations of the word superiority effect, researchers have also shown a pseudoword superiority effect: letters are more easily identified in pronounceable non-words (henceforth referred to as pseudowords to distinguish from unpronounceable non-words) than letters alone (e.g., McClelland and Johnston, 1977) or letters in non-word contexts (e.g., Baron and Thurston, 1973; Spoehr and Smith, 1975). Given that difficulty pronouncing pseudowords is one of the identifying characteristics of developmental dyslexia (for review, see Rack et al., 1992), one might predict that there would be a more dramatic difference between those with dyslexia and controls in the magnitude of a pseudoword superiority effect. Nonetheless, Grainger et al. (2003) also found no difference between groups on the pseudoword superiority effect: The effect was present in both the children with dyslexia and the reading-age matched controls and the magnitude was roughly the same in both groups. Hence, any explanation of the differing ability to pronounce pseudowords cannot depend solely on processes involved in the pseudoword superiority effect. In particular, Grainger et al. claim that this finding rules out the common explanation of dyslexia as a deficit in letter (or letter clusters) to phoneme translation.

A third finding in the Grainger et al. work was that, with both dyslexic and control groups of children, there was no difference in the magnitude of the word superiority effect and of the pseudoword superiority effect. That is, the increase in performance for letters in words over letters in isolation was roughly the same size as the increase in performance for letters in pseudowords over letters in isolation. In contrast, the normal-reading adults in their study had a larger advantage for word context compared to pseudoword context, a difference that has been found in many other studies (Manelis, 1974; McClelland and Johnston, 1977; Estes and Brunn, 1987; Jacobs and Grainger, 1994).

Houpt et al. (2014) recently demonstrated a new approach to measuring the word superiority effect based on response times to whole letter strings rather than accuracy of single letter identification. Their approach is based on a comparison of an individuals response latency to a full string, such as a word or pseudoword, to his predicted response time if he had identified each letter independently and in parallel. This method has multiple potential advantages for studying word superiority among those with dyslexia. First, it is an individualized measure so we can study both differences across groups as well as heterogeneity within those with dyslexia. Second, even though compensated dyslexic adults may increase word recognition and accuracy, reading is often still less automatic, fluid, and fast (Lefly and Pennington, 1991; Shaywitz et al., 1999), so the fact that the Houpt et al. approach is based on response times may make it more likely to pick up on differences between the groups. Finally, it is a model based approach, so the results can inform models of word perception by both normal-reading adults and those with dyslexia.

The main statistic used by Houpt et al. (2014) was the capacity coefficient (Townsend and Nozawa, 1995; Townsend and Wenger, 2004; Houpt and Townsend, 2012), which uses the cumulative reverse hazard function of the response times to predict hypothetical independent, parallel performance and compare it to participants actual performance. For more details see (Houpt et al., 2014). For each participant the cumulative reverse hazard function is estimated from single character conditions by the sum over all response times less than a given time of 1/number of response times less than or equal to *t*, i.e.,

K(t)=1/n∑1/Y(t).

The independent parallel model prediction for a participant is given by summing the cumulative reverse hazard functions over each of the characters (Townsend and Wenger, 2004; Houpt et al., 2013). The participants actual performance with words (or pseudowords, etc.) is then compared to the predicted independent, parallel performance to get a measure of the degree of the advantage or disadvantage of the context.

$$C(t)=K_{\text{L}etter1}+K_{\text{L}etter2}+K_{\text{L}etter3}+K_{\text{L}etter4}-K_{\text{word}}$$

When the capacity coefficient is positive, indicating participants performed better with context, it is referred to as super-capacity. If the capacity coefficient is negative, which occurs if participants perform worse, it is referred to as limited capacity. Finally, if their performance is approximately equal to the predicted independent parallel model, it is referred to as unlimited-capacity.

The participants reported in Houpt et al. (2014), who had no reported reading difficulties, were nearly all super-capacity with words and pseudowords, while they tended to be limited-capacity with unpronounceable non-words and were nearly all limited capacity with upside-down, unpronounceable, non-words and unfamiliar characters (Katakana). They found that words and pseudowords were higher capacity than the other string-types. However, unlike the larger advantage for words over pseudowords normally reported (including for adults in Grainger et al., 2003), they only found higher capacity for words compared to pseudowords when the stimuli were not masked.

There are multiple potential outcomes to applying the capacity approach to analyzing dyslexia. If the time based measures follow the accuracy based results of Grainger et al., then we would expect to see super-capacity for words and pseudowords and unlimited or limited capacity for non-words for both dyslexic and control participants. With normal reading adults, we would also expect to see higher capacity with words than with pseudowords, although this prediction is less certain given that Houpt et al. only found the difference in capacity in one of their two experiments. If the deficits present in dyslexia are specific to word perception speed, but not accuracy, then we would expect word and pseudoword capacity to be unlimited or limited, more on par with non-word capacity. We would also predict that the participants with dyslexia would have generally lower capacity with words and pseudowords than the control group.

## 2. Method

To measure the cumulative hazard function for responses to strings, we had a block of trials dedicated to each string type in which the same target and distractors were used. Targets were all four character strings: “care” for the word blocks, “lerb” for the pseudoword blocks, “rlkf” for the non-word blocks and “
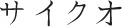
” for the unfamiliar character blocks. For each target, a set of four distractors was chosen that was within the same category, e.g., all of the distractors for the word-target block were also words. Each distractor was created by changing a single character in the target string, with one distractor for a change in each character position, e.g., for the target “rlkf,” the distractors were “vlkf,” “rtkf,” “rlhf,” and “rljk.” This is essentially the same task as Houpt et al. (2014).

To measure the cumulative hazard function for characters in isolation, we had blocks of trials in which participants needed to discriminate between each of the two possible characters in each position. For example, because “vlkf” was a distractor for the target “rlkf,” we had a block of trials during which the participants were required to distinguish between “v” and “r” in isolation. The full set of stimuli we used are shown in Table 1.

**Table 1:** **Full set of character sequences used for stimuli**.

	**Target**	**Distractors**				**Single character**							
Word	care	bare	cure	cave	card	c	b	a	u	r	v	e	d
Pseudoword	lerb	nerb	larb	lemb	lerf	l	n	e	a	r	m	b	f
Non-word	rlkf	vlkf	rtkf	rlhf	rljk	r	v	l	t	k	h	f	k
Katakana	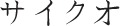	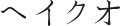	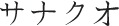	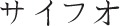	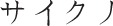	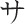	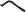	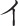	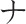	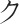	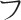	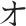	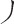

### 2.1. Participants

Participants were 19 students (Mean age = 21; 15 female) recruited from the Indiana University community. 11 participants had a formal dyslexia diagnosis and one dyslexia participant was left handed. Two of the participants with dyslexia (both Male) were dropped from the analyses because they did not complete 2 days of each of the experimental sessions. All control participants had no history of neurological conditions. All participants provided written informed consent, as approved by the Institutional Review Board of Indiana University, Bloomington. The participants completed a battery of tests to measure cognitive performance. They completed the Wechsler Abbreviated Scale of Intelligence (WASI; Weschler, 1999), Word Attack (pseudoword naming) from the Woodcock-Johnson III tests of Achievement (Woodcock et al., 2001), the Edinburgh Handedness Questionnaire (Oldfield, 1971), Dyslexia Checklist (Vinegrad, 1994), and the Adult Reading History Questionnaire (Lefly and Pennington, 2000). As shown in Table 2, the groups did not differ on intelligence measurements, but did differ on measures of phonological processing and verbal working memory. Also, although all but one participant reported being right handed, the groups differed in degree of handedness with the dyslexics having a weaker absolute handedness measure.

**Table 2:** **Descriptive measures of participant groups**.

	**Control (**n** = **8**)**		**Dyslexia (**n** = **9**)**		***t***	**BF**
	***M***	***SD***	***M***	***SD***		
Age	21.5	(2)	21.3	(1.3)	0.20	0.43
WASI verbal	113	(12.6)	118	(10.9)	−0.87	0.55
WASI non-verbal	105.4	(5.5)	110.7	(10.3)	−1.34	0.73
WASI full	110.5	(9.6)	115.6	(11.3)	−1.00	0.58
Verbal - Non-verbal	7.6	(9.2)	7.33	(9.34)	0.06	0.42
Handedness	81.8	(17.6)	57.9	(19.9)	2.63	3.23
Dyslexia checklist	4	(3.3)	14.9	(3.3)	−6.79	2120
Reading history	29.9	(11.2)	67.3	(10.3)	−7.14	3650
Reading span	3.1	(0.7)	2.1	(0.22)	3.55	17.4
Word attack (GE)	15.26	(3.7)	7.36	(1.95)	4.92	159

BF refers to the Bayes Factor comparing a model in which there is a difference between groups to a model in which there is no difference between groups. BF larger than 1 indicates evidence in favor of a difference with >3.2 considered substantial evidence, >10 strong and >100 decisive. BF below 1 indicates evidence in favor of no difference between groups (<0.31 substantial, <0.1 strong, <0.01 decisive).

Groups did not differ in age or intelligence measures. On average, verbal IQ was higher than non-verbal IQ (*M* = 7.26, *SD* = 9.63, *p* < 0.005), but this did not differ by group.

### 2.2. Stimuli

Table 1 gives the complete list of stimuli used for both the single character and exhaustive trials for each type, which are a subset of the stimuli in Houpt et al. (2014). There were four categories of stimuli used: words, pronounceable non-words (pseudowords), unpronounceable non-words and strings of Katakana characters. All strings used were four characters long. Word frequency counts (based on Kucera and Francis, 1967) are available in the appendix of Houpt et al. (2014). Pseudowords were taken from the ARC Non-word Database (Rastle et al., 2002). The neighborhood size and summed frequency of the neighbors for each of the pseudowords are also included in the appendix of Houpt et al. (2014). Strings and characters were presented in black Courier font on a gray background. Characters were approximately 0.33° horizontally and between 0.30° and 0.45° vertically. Strings were about 1.5° horizontally.

### 2.3. Procedure

All experimental conditions were run using Presentation°ledR software version 14.9 (www.neurobs.com). Stimuli were presented on a 17″ Dell CRT monitor running in 1280 × 1024 mode. Participants used a two-button mouse for their responses. Participants were paid $8 per session, and received a $20 bonus upon completion of all 10 sessions. Each session lasted between 45 and 60 min. The first session was dedicated to general cognitive and reading ability assessment. The second through ninth sessions were each dedicated to one of the four stimulus types (e.g., word, pseudoword, …), so there were two sessions of each type. The order of string-types was randomized across participants. At the beginning of each session, we read the participant the general instructions for the task while those instructions were presented on the screen. The instructions encouraged participants to respond as quickly as possible while maintaining a high level of accuracy. Each session was divided into five blocks, one block of string stimuli and a block for each of the corresponding single character stimuli. The final session was a dedicated EEG session, although those data are not further discussed here.

Each block began with a screen depicting the button corresponding to each of the categories. Participants first completed 30 practice trials of the stimulus type in that block. Next, participants completed 170 trials. Half of the trials were with the target stimulus and the other half were divided evenly among the distractor set. Each trial began with a 500 ms presentation of the block instruction screen which included a diagram of a computer mouse that depicted which button to press for the target and distractors, respectively. One button of the mouse was associate with the target string (e.g., “care”) and the other button was associated with the distractor(s) (e.g., “bare,” “cure,” “cave,” and “card”). In the single character trials, there was only one stimulus associated with each button (e.g., left button: “c”; right button: “b”). The instruction screen was followed by a 500 presentation of a fixation cross. The stimulus was then presented for 100 ms. Participants had a maximum of 1600 ms to respond. Participants did not receive feedback about the correctness of their response. The session order was counterbalanced among the participants so that participants completed the different types on different days and in different orders.

### 2.4. Analysis

All data were analyzed using R statistical software (R Development Core Team, 2011). We computed Bayesian ANOVA of the correct target response times using the BayesFactor package (Rouder et al., 2012). The Bayes factor (BF) approach to ANOVA uses model comparison to give evidence for or against including independent variables as predictors for the dependent variables. The BF indicates the ratio of posterior probability of observed data given the model for a pair of models. A rough scale for interpretation of the BF is as follows: <0.01 decisive evidence against; <0.1 strong evidence against; 0.31 substantial evidence against; 0.32–1; minimal evidence against; 1–3.2 is minimal evidence for; >3.2 substantial evidence for; >10 strong evidence for; >100 decisive evidence for (Jeffreys, 1961). Capacity analyses were completed using the sft package (Houpt et al., 2013).

## 3. Results

### 3.1. Mean response time and accuracy

For each analysis, we computed the Bayes Factor for a full model, which included string-type (word, pseudoword, random, or Katakana), target/distractor, day (1 or 2), and group (control or dyslexia), relative to a subject intercept only model. We then compared that Bayes factor to successively simpler models which were derived by first removing interactions terms then main effects while maintaining a component for any lower order effects that were included in an interaction term.

**Figure 1:**
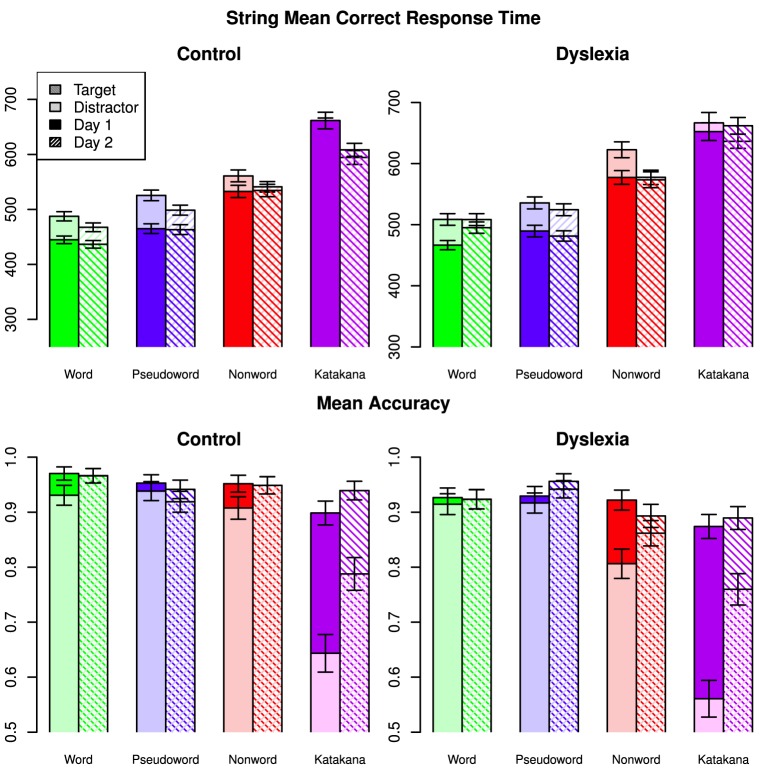
**Mean correct response times and mean accuracies for all string types across days, targets/distractors and group**. Error bars indicate 95% confidence intervals.

Accuracy and mean correct response times with the string blocks for each string-type are shown in Figure 1 with error bars representing the 95% credible intervals from the full model. The highest Bayes factor model for correct response times included a three-way interaction among string-type, day and group along with two-way interactions between string-type and target/distractor and day and target/distractor. This model had a Bayes factor of 19.9 (strong evidence) over the next best model, which included a group by target/distractor interaction and was otherwise the same. There was decisive evidence for the best model over all other models (*BF* > 125).

Analysis of the posterior of the full model indicated that the three-way interaction was driven by the control group speeding up on Katakana on Day 2 compared to Day 1, while the dyslexia group was relatively faster on non-words on Day 2 compared to Day 1. The string-type by target/distractor interaction was driven by a cross-over targets being slower for words and pseudowords and faster for Katankana. The string by day interaction, marginalized across group, showed a cross-over between faster performance for words on Day 1 relative to Day 2 and slower performance for Katakana on Day 1 relative to Day 2. A marginal interaction between string-type and group was mostly driven by faster performance by the controls on the non-word stimuli.

Marginalized over the other factors, words were faster than pseudowords (Posterior Mean = 20.7, 95% HDI = [15.6, 25.7]), non-words (Posterior Mean = 89.3, 95% HDI = [84.1, 94.6]), and Katakana (Posterior Mean = 164, 95% HDI = [159, 170]). Additionally pseudowords were faster than non-words (Posterior Mean = 68.7, 95% HDI = [64.4, 73.4]) and Katakana (Posterior Mean = 144, 95% HDI = [138, 149]) and nonwords were faster than Katakana (Posterior Mean = 74.9, 95% HDI = [69.6, 80.3]). Targets were slower than distractors (Posterior Mean = −20.1, HDI = [−23.6, −16.6]). Response time on Day 1 were slower than on Day 2 (Posterior Mean = −14.7, HDI = [−18.6, −10.9]). There was not clear evidence for one group being faster than the other overall (Posterior Mean of Control minus Dyslexia = −25.4, HDI = [−106, 54.5]).

The highest Bayes Factor model for accuracy included a three-way interaction among string-type, day and target/distractor and a two-way interaction between string-type and group. There was strong evidence for this model over a model which also included a group by target interaction (*BF* = 12.0) and over a model that included a group by day interaction (*BF* = 29.8). There was decisive evidence for the best model over all other models (*BF* > 159).

The three-way interaction in accuracy comes from the large increase in distractor performance across days on Katakana and a slight increase in performance for distractors relative to target on words and non-words compared to a unchanged relative performance on the pseudowords across days. Overall there was a larger increase in performance for Katakana than the other string-types, with the smallest changes in the word and pseudoword blocks. Between groups, there was a larger difference in accuracy in the non-word blocks and the smallest difference for pseudowords. Between targets and distractors, the largest difference was for Katakana and the smallest differences were for the word and pseudoword string-types. Generally, distractor performance improved more between the days than target performance.

Marginalized over the other factors, accuracy with words was nearly the same as accuracy on pseudowords (Posterior Mean = 0.00323, 95% HDI = [−0.00667, 0.00129]), slightly better than non-words (Posterior Mean = 0.0351, 95% HDI = [0.0254, 0.0448]), and much better than Katakana (Posterior Mean = 0.146, 95% HDI = [0.136, 0.156]). Additionally pseudowords were slightly more accurate than non-words (Posterior Mean = 0.0318, 95% HDI = [0.0219, 0.0416]) and much more accurate than Katakana (Posterior Mean = 0.143, 95% HDI = [0.133, 0.153]) and non-words were more accurate than Katakana (Posterior Mean = 0.111, 95% HDI = [0.101, 0.121]). Targets were more accurate than distractors (Posterior Mean = 0.0724, HDI = [0.0654, 0.0794]). Accuracy on Day 2 was higher than on Day 1 (Posterior Mean = 0.0326, HDI = [0.0256, 0.0395]). There was not clear evidence for one group being more accurate than the other overall (Posterior Mean of Control minus Dyslexia = 0.0353, HDI = [−0.0476, 0.117]).

**Figure 2:**
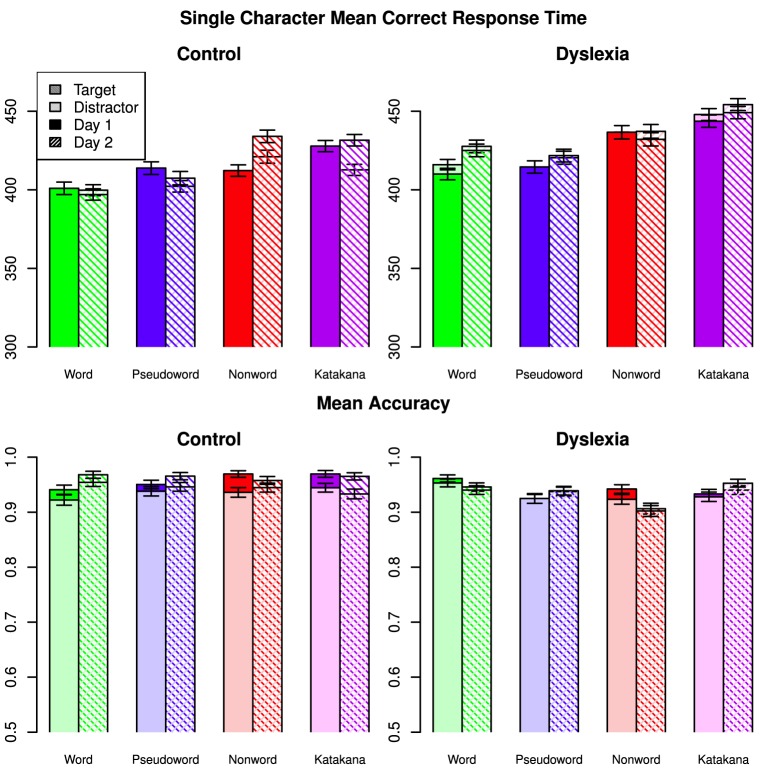
**Mean correct response times and mean accuracies on single characters for all types across days, targets/distractors and group**. Error bars indicate 95% confidence intervals.

Mean correct response time and accuracy with the single character blocks for each type are shown in Figure 2.

As in the string data, the best model included a three-way interaction among character-type, day and group. There were also two-way interactions between character-type and day, character-type and group, day and group, and group and target/distractor. There was very strong evidence for this model over the next best, which also included a character-type by target/distractor interaction (*BF* = 65.7), and the third best model which included a day by target/distractor interaction (*BF* = 81.2). There was decisive evidence for the best model over all others (*BF* > 2600).

The three-way interaction was driven by the slow-down for control participants on the non-word task between days, while there was no such change for the dyslexia group. The character-type by day interaction was also mainly due to the slow down on the non-words between days. The control group was relatively faster on words and Katakana, while there was a smaller group difference on the non-word characters and nearly no group differences on the characters from pseudowords. The control group slowed down less from Day 1 to Day 2 than the dyslexia, although the magnitude of this difference was small. Control participants were had a relatively larger speed up for distractors over targets on Day 1 than dyslexia participants compared to the second day.

There was a small response time advantage for word characters compared to pseudoword characters when the other factors were marginalized (Posterior Mean = −2.89, HDI = [−4.73, −1.00]) and large advantages for word characters over non-word characters (Posterior Mean = −17.5, HDI = [−19.3, −15.6]) and Katakana characters (Posterior Mean = −26.1, HDI = [−27.9, −24.2]). Pseudoword characters were faster than non-word characters (Posterior Mean = −14.6, HDI = [−16.5, −12.7]) and Katakana characters (Posterior Mean = −23.2, HDI = [−25.0, −21.3]). Non-word characters were faster than Katakana characters (Posterior Mean = −8.55, HDI = [−10.4, −6.67]). The marginal group response times were again indistinguishable (Posterior Mean = −15.6, HDI = [−74.0, 40.7]).

For the single character accuracy data, the best fit model again included the three-way interaction among character-type, day and group. There was decisive evidence for this model over all alternative models (*BF* ≥ 138). There was a small advantage for word characters over pseudoword characters (Posterior Mean = 0.00756, HDI = [0.00372, 0.0115]) and non-word characters (Posterior Mean = 0.0129, HDI = [0.00906, 0.0168]) but not a clear difference between word and Katakana characters (Posterior Mean = 0.00239, HDI = [−0.00146, 0.00624]). Participants were slightly more accurate characters with pseudoword characters than non-word characters (Posterior Mean = 0.00531, HDI = [0.00138, 0.00918]) but less accurate with pseudoword characters compared to Katakana characters (Posterior Mean = −0.00517, HDI = [−0.00905, −0.00126]). Participants were also slightly less accurate with non-word characters than with Katakana characters (Posterior Mean = −0.0105, HDI = [−0.0143, −0.00658]). There were no clear marginal differences between days (Posterior mean of Day 2 minus Day 1 = 0.00232, HDI = [−0.000389, 0.00505] or groups (Posterior mean of Control minus Dyslexia = 0.0148, HDI = [−0.0494, 0.0789]).

Because response time distributions tend to be skewed, and these data are no exception, we also ran an analysis on the log-transformed response time data and found no difference in which model had the highest Bayes factor and only a small difference in the magnitude of that Bayes factor compared to the next best model for the string data (*BF* = 17.8) and resulted in stronger evidence for the character data (*BF* = 217).

### 3.2. Capacity analyses

**Figure 3:**
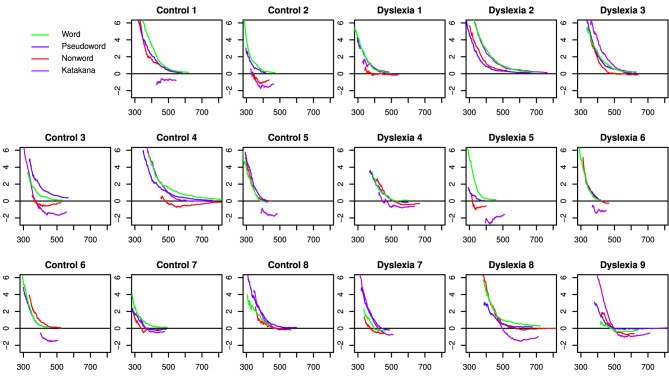
**Difference capacity coefficients for each participant in each string type, collapsed across days**. Under the null-hypothesis of unlimited-capacity, independent, parallel character recognition, the function would be equal to zero for the full time range.

**Figure 4:**
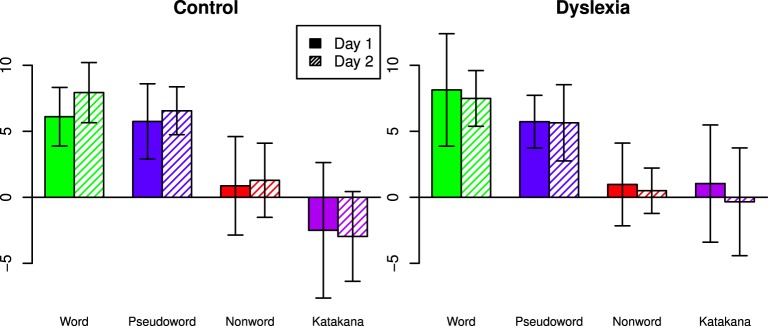
**Mean capacity statistic values across days, string-type and group**. Under the null-hypothesis of unlimited-capacity, independent, parallel character recognition, the statistic would be have a standard normal distribution at the individual level. Error bars indicate 95% confidence intervals.

Capacity coefficients are shown for each individual (collapsed across days) in Figure 3. Using the capacity statistic from Houpt and Townsend (2012), participants tended to be super-capacity in the Word (Control: Day 1 = 7/8, Day 2 = 8/8; Dyslexia: Day 1 = 7/9, Day 2 = 7/8 significantly better than baseline) and Pseudoword string-types (Control: Day 1 = 7/8, Day 2 = 8/8; Dyslexia: Day 1 = 9/9, Day 2 = 7/8 significantly better than baseline). Figure 4 summarizes the overall capacity statistic for each group on each day. There was more variable performance with Katakana (Control: Day 1 = 2/8 above and 5/8 below, Day 2 = 1/8 above and 5/8 below; Dyslexia: Day 1 = 3/9 above and 3/9 below, Day 2 = 3/9 above and 4/9 below) and the non-words (Control: Day 1 = 3/8 above and 2/8 below, Day 2 = 2/8 above and 2/8 below; Dyslexia: Day 1 = 4/9 above and 4/9 below, Day 2 = 1/8 above and 2/8 below).

The best model based on a Bayesian ANOVA measuring day, group and string-type predicting the individual capacity *z*-scores included a group by string-type interaction as well as main effects of group and string-type. The evidence was nearly equivocal when compared to a model with only a main effect of string-type (*BF* = 1.73) but had at least substantial evidence over all other models (*BF* ≥ 4.00). Table 3 shows the Bayes Factor for the best model relative to all models over which there was not very strong or decisive evidence.

**Table 3:** **Bayes Factors for the highest model relative to the next best models for predicting capacity z-scores**.

**Model**	***BF***
String-type + Subject	1.00
String-type + Group + Subject	0.417
String-type + Day + Subject	0.194
String-type × Group + String-type + Group + Subject	0.181

Capacity z-scores were close between words and pseudowords (Posterior Mean = 1.44, HPD = [−0.367, 3.19]) and higher for words than non-words (Posterior Mean = 6.29, HPD = [4.49, 8.10]) and Katakana (Posterior Mean = 8.35, HPD = [6.56, 10.1]). Pseudoword capacity z-scores were higher than both non−words (Posterior Mean = 4.86, HPD = [3.06, 6.64]) and Katakana (Posterior Mean = 6.91, HPD = [5.08, 8.75]). Non-words had higher capacity z-scores than Katakana (Posterior Mean = 2.06, HPD = [0.292, 3.82]). There was nearly no marginal difference between groups (Posterior Mean = −0.526, HPD = [−3.16, 2.04]).

The capacity z-score gives a summary of the capacity function across time. To check for differences in the shape of capacity coefficient functions, we tested the factor scores obtained from functional principal components analysis (fPCA) of the capacity coefficients (Burns et al., 2013). fPCA is a dimensionality reduction technique that is essentially the same as the more familiar principal components analysis for vectors. The main difference in fPCA is that the data are described in terms of a linear combination of *functions* rather than vectors.

**Figure 5:**
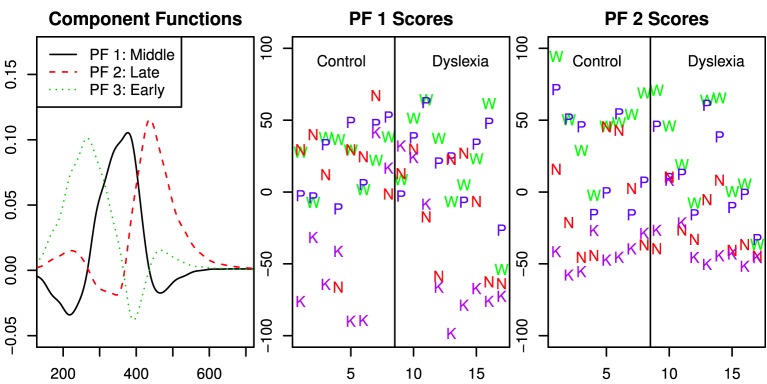
**Functional principal components analysis of the capacity functions across all participants and stimulus types**. The first panel shows the component functions after the varimax rotation. The second and third panels show the scores for the first and second component function. The scores are separated for the control group and those with dyslexia, however the fPCA solution was computed for all data together.

Because the best model of capacity effects did not include day and better estimates of capacity functions lead to more accurate principal component representation, these analysis were performed with data collapsed across day. The fPCA indicated that the variation across capacity functions was well-represented by three factors related to early, middle and late response time regions (see Figure 5).

According to the Bayes factor analyses reported in Table 4, there was clear evidence of variation in the capacity functions due to string type in the middle and late time regions. Evidence was present, but less clear, against an effect of group. The analysis was nearly equivocal with respect to meaningful variation in the early time region beyond the variation due to individual subject.

**Table 4:** **Bayes Factors relative to the highest model for predicting fPCA capacity scores**.

**Model**	**Middle (D1)**	**Late (D2)**	**Early (D3)**
String-type + Subject	1.00	1.00	1.00
String-type + Group + Subject	0.437	0.633	0.345
String-type × Group + String-type + Group + Subject	0.131	0.270	0.083
Group + Subject	2.96×10^−6^	8.25×10^−11^	0.194
Subject	7.83×10^−6^	1.83×10^−10^	0.580

A visual inspection of the individual participant capacity plots in Figure 3 suggest different patterns of results across string-types for different participants. First, some participants showed much higher capacity for words and pseudowords than for Katakana, with lower capacity for non-words, but not as low as Katakana (e.g., Controls 1, 2, and 3 and Dyslexia 5). This is basically the pattern of results reported in Houpt et al. (2014). Another set of participants had mostly similar capacity functions across string-type (e.g., Controls 7 and 8 and Dyslexia 9).

**Figure 6:**
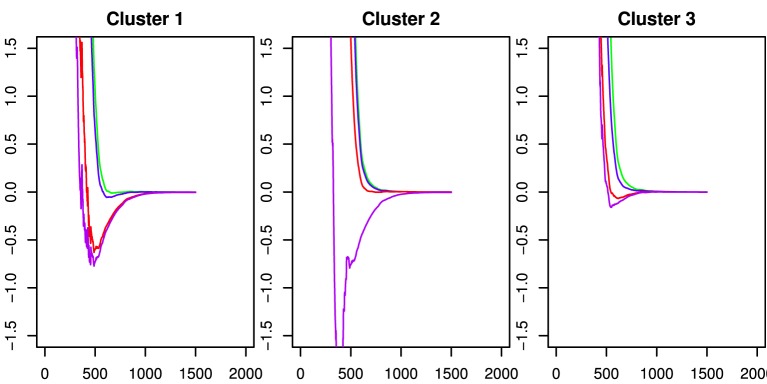
**Capacity functions representing the center of each of the three k-means clusters**. These are derived by using the mean vector of the cluster on the fPCA scores to as factor weights to determine the functions. The colors indicate the string types using the same scheme as the preceding figures. Word: Green; Pseudoword: Blue; Nonword: Red; Katakana: Purple.

To investigate these patterns of differences and the extent to which they may be predictive of the basic behavioral measures, we used *k*-means clustering on the fPCA scores. Inspection of a scree plot indicated three clusters would be appropriate for these data. The capacity functions represented by the three cluster means are shown in Figure 6. The pattern in Cluster 2 is most similar to the results in Houpt et al. (2014) whereas Cluster 3 represents the participants who had less variation in capacity across string-type. Similar to Cluster 2, Cluster 1 had higher capacity for words and pseudowords and limited capacity for Katakana, but Cluster 2 also had fairly limited capacity for non-words. Control participants were all in either Cluster 2 (4/8) or Cluster 3 (3/8) except Participant C4, who was in Cluster 1. Four of the nine Dyslexia participants were in Cluster 1, three in Cluster 3 and two in Cluster 2. Note that neither dyslexia status nor the reading and cognitive performance measures contributed to discovering the clusters.

**Figure 7:**
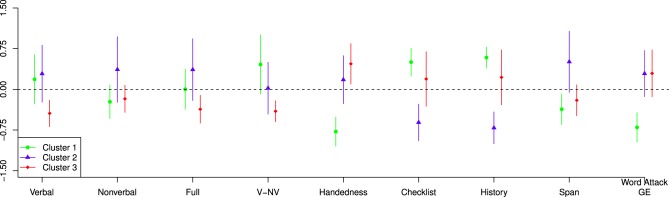
**Representation of the variation in the diagnostic tests across the clusters which were derived from the capacity analysis**. The points (triangles, squares, and circles) represent the mean value and the lines represent the standard error of the mean.

Probing deeper into the connection between the capacity task and the reading and cognitive task, we also examined the variation in those measures across clusters. Figure 7 shows the distribution (after standardizing across participants) of the basic behavioral measures across each cluster. Generally speaking, Cluster 1 was distinguished in these measures by being have lower handedness scores and lower scores on the Grade Equivalent Word Attack; Cluster 2 had lower Dyslexia checklist scores, higher reading span scores and lower reading history scores; and Cluster 3 had slightly lower verbal IQ scores. Despite the pattern of differences across the measures, Bayesian ANOVAs did not indicate strong evidence either for or against differences among the clusters on any single measure (0.4 ≤ BF ≤ 2.5 due to the small number of participants in the study.

## 4. Discussion

In the current study we aimed to explore word perception differences in dyslexia using a novel approach, capacity measures designed to investigate response time latencies. We compared participants with dyslexia and age-matched controls on a discrimination task with four types of stimuli: words, pseudowords, non-words, and Katakana.

The lack of a marginal level difference in either response time, accuracy or capacity based on dyslexia diagnosis replicates and extends the basic finding of Grainger et al. (2003) and the replication in Ziegler et al. (2008): Word superiority effects are present at a group level for those with a dyslexia diagnosis and at a similar magnitude to age-matched control groups. This finding is extended in this paper to a new group, college aged students, and a new paradigm, the design from Houpt et al. (2014).

However, in our current study, the response latency showed a three-way interaction between group, string-type, and day, suggesting that there are some subtle differences between controls and dyslexics. Additionally, the mean capacity results were similar to those found in a previous study by Houpt et al. (2014) using this technique—words and pseudowords had similarly higher capacity than non-words and non-words had higher capacity than Katakana. Interestingly, when the capacity results were inspected, individual differences emerged such that three different capacity profiles emerged. One group was similar to the non-dyslexics reported in the Houpt study while the other two groups had capacity profiles that diverged in important ways.

The *k*-means clustering analysis indicated three distinct capacity profiles. In an attempt to characterize these three profiles we also explored the cognitive/behavioral scores of the individuals that composed them. The profile that most resembled (Houpt et al., 2014), Cluster 2, had scores more similar to those expected of normal reading adults (i.e., lower dyslexia checklist and reading history scores and higher reading span scores). Indeed, the two dyslexic participants whose capacity profiles were included in Cluster 2 had the lowest dyslexia checklist and reading history scores among those with dyslexia.

Like Cluster 2, the capacity profile for Cluster 1 showed high capacity for words and pseudowords and lower capacity for Katakana, but also showed lower capacity for non-words that was similar to Katakana. The individuals that made up Cluster 1 on average had low Word Attack scores and reading span scores, and high reading history and dyslexia checklist scores, all of which are indicative of dyslexia. The one control participant who was included in this cluster had the highest dyslexia checklist and reading history scores. Interestingly, with the exception of one dyslexic in Cluster 3, the dyslexics in Cluster 1 showed the lowest Word Attack Grade Equivalent scores (all below 7th grade) and the members of this group appear to show an efficiency divide between pronounceable and non-pronounceable string-types. Low performance on Word Attack, particularly in college students, may suggest that the grapheme-to-phoneme processes for this group are particularly affected. This may prompt a “whole word” strategy when reading. They do appear to be efficient in visually recognizing whole regular words and whole pseudowords. The efficiency for pseudowords may be due to repetition causing them to be processed more like words. Although the participants in Cluster 1 had low Word Attack scores, the pseudowords in this study were four-letter, single-syllable pseudowords that are relatively easy to pronounce. Therefore, they may have treated pseudowords like words once they were learned (e.g., on day 2). However, this may not be possible for non-pronounceable consonant strings or foreign characters because they were unable to be learned as words (e.g., non-words are orthotactically invalid and Katakana is not linguistically meaningful). A study by Siegel et al. (1995) suggested that dyslexics with low phonological awareness rely more on orthographic processing. Specifically, they noted a group of dyslexics with poor performance on Word Attack, but high orthographic awareness compared to controls with higher Word Attack scores.

The final profile identified by *k*-means clustering, Cluster 3, revealed little differences among the four stimulus types. The individuals who showed this profile included both dyslexic and control participants. In terms of test scores, only Word Attack and verbal IQ differentiate Cluster 3 and Cluster 1. On average, individuals in Cluster 3 had higher Word Attack scores and lower verbal IQ. This suggests that these individuals may not have a weaknesses related to grapheme-to-phoneme conversion, but may have deficits in other language-related processes that account for the lower verbal IQ. The finding that the capacity scores were similar across stimulus types suggests that individuals in Cluster 3 used a generalized strategy. Because all participants were naive to Katakana, a generalized strategy could not have depended on linguistic processing but may instead have depended on visual feature processing. This strategy is apparently very efficient and able to handle complex unfamiliar visual stimuli. It is possible that this is a global, holistic process. Some evidence to support such a strategy comes from a study examining high school students that found dyslexics were faster, but not more accurate, at detecting impossible objects (von Károlyi, 2001). They found that these students relied on global processing of the objects (e.g., recognizing features simultaneously and discerning if they contradict each other). While Katakana does not have any inherent contradictory features in this study, if we situate the target Katakana string as the goal this contextualizes the distractor strings as somewhat contradictory. It is possible that the participants who were efficient at Katakana (as well as the other string-types) were processing the strings as whole objects. It is also possible that many of the dyslexic members of Cluster 3 were especially good at Katakana because language processing could not “get in the way.” They may then have been able to generalize a visual, non-linguistic strategy into the other categories.

While it may be that individuals in Cluster 3 used a non-linguistic strategy, an alternative explanation is that a linguistic strategy was used for non-word and Katakana stimuli. In an MEG study of visual word recognition in dyslexia, Salmelin et al. (1996) found that non-dyslexics displayed a typical sharp negativity around 180 ms in temporo-occipital regions to words, but dyslexics only activated this region after 200 ms with a slowly increasing signal that peaked closer to 450 ms. Some of the participants in the current study also participated in an pilot EEG session of the task after completing the study. Generally, participants who showed a profile similar to Cluster 3 failed to show a sharp left N170 in response to the stimuli, but instead showed a more gradual negativity in less lateralized posterior electrodes that peaked between 220-350 ms; this pattern was fairly consistent across string-type (Sussman et al., 2011). In contrast, a control with a non-clumping capacity pattern, similar to Cluster 2, showed a more typical pattern of an N180 in left temporo-occipital electrodes for words, pseudowords, and non-words; but for Katakana did not show this N180 response. The correspondence between our EEG data and (Salmelin et al., 1996) potentially suggests that the participants who show similarity in capacity across all four string-types are generalizing a strategy from words to Katakana and not vice versa. This also suggests, however, that the presumed compensatory strategy they are using requires visual language processes. Interestingly, the Cluster 3 pattern is not unique to the dyslexia participants and was, in fact, used by some controls. That most (all three dyslexics and one control) of the subjects in Cluster 3 showed super-capacity for Katakana suggests that the strategy was more generalized across string-types, but not always efficient. It is possible that particularly the dyslexics in this group are more practiced at using a generalized strategy. Further research is necessary to determine the strategy being used by individuals in Cluster 3.

Together with the results from Grainger et al. (2003) and Ziegler et al. (2008), these results indicate that there is no general deficit in orthographic recognition, either at the single character or configural level, with dyslexia. Some of the participants with dyslexia were differentiated from most of the control participants in this task, but the main difference was in their performance on non-words. Given the low Word Attack scores, it is unlikely that the participants with dyslexia are using phonological information for better performance in the word and pseudoword condition, so they are potentially relying on information from the orthographic configurations. The subgroup that performed worse on non-words may have relied more on statistical regularities in letter combinations (cf. Pelli et al., 2003) than the participants who were not much worse with non-words. Although previous research has shown that the effect of orthographic regularity across languages (English and German) is similar across participants with and without dyslexia (Landerl et al., 1997), in future research it would be worthwhile to investigate whether there is a difference in the effect of orthographic regularity associated with the different capacity profiles reported herein.

One potential limitation of the current study, and of the approach in Houpt et al. (2014), is that only a single string is used for each string-type. In the standard Reicher–Wheeler paradigm, a different word is used on each trial. Because the repeated presentation of the string, there is ample opportunity for the participants to use encoding strategies that are efficient for those particular strings, but are not necessarily representative of the participants' ability across the whole class of string-types that is represented by that string. Despite this possibility, Houpt et al. (2014) found a clear differentiation among the string types. Although it is more parsimonious to assume, that the same perceptual process differences underly the word and pseudoword superiority effects observed in both (Houpt et al., 2014) and the Reicher-Wheeler design, it leaves open the possibility that the individual differences in this study were due to differences in perceptual learning rather than differences in more general, stable, perceptual encoding strategies.

Another limitation of the current work is that the participants were undergraduate and graduate students at a major university. These participants may not be representative of the wider range of adults with dyslexia. Furthermore, these participants have had many years of reading practice to develop strategies for ameliorating the effects of dyslexia. In future work, it would be informative to use this paradigm with younger children who have not had access to as many years of remediation training as the participants in this study. This would facilitate further connection between the effects reported here and the previous studies of dyslexia and the word superiority effect (Grainger et al., 2003; Ziegler et al., 2008). It would be particularly interesting to test if the same clusters of capacity performance emerge with younger participants or perhaps if there is some effect of remediation training on the capacity patterns. More generally speaking, this is a relatively small sample of participants for individual differences research and we hope to expand these results to a much larger sample.

To conclude, the results presented here emphasize the importance of exploring individual differences. The dyslexic group, like the control group, is not homogeneous; they do not all process word and word-like strings in the same way. Here, when examining capacity profiles, three different subgroups were observed and there were both control and dyslexic participants in each of these groups. While it is difficult to detect these patterns by only examining the accuracy data from tasks designed to explore the word superiority effect (e.g., Grainger et al., 2003), by using response latency data to predict independent, parallel processing, group differences emerged. These types of analyses may prove to be informative and provide information regarding how individuals are processing word stimuli, which can then be used to develop remediation tools that are tailored to an individual dyslexic.

### Conflict of interest statement

The Guest Associate Editor Cheng-Ta Yang declares that, despite having collaborated with authors James T. Townsend and Joseph W. Houpt, the review process was handled objectively and no conflict of interest exists. The authors declare that the research was conducted in the absence of any commercial or financial relationships that could be construed as a potential conflict of interest.
